# Study of a US cohort supports the role of *ZNF644* and high-grade myopia susceptibility

**Published:** 2012-04-12

**Authors:** Khanh-Nhat Tran-Viet, Elizabeth St.Germain, Vincent Soler, Caldwell Powell, Sing-Hui Lim, Thomas Klemm, Seang Mei Saw, Terri L. Young

**Affiliations:** 1 The Center for Human Genetics, Duke University Medical Center, Durham, NC; 2 UMRS 563, Centre de Physiopathologie de Toulouse Purpan, Université Paul Sabatier, Toulouse, France­­­­­­­; 3 Saw Swee Hock School of Public Health, National University of Singapore, Singapore; 4 Duke-National University of Singapore Graduate Medical School, Singapore; 5 Singapore Eye Research Institute, Singapore; 6 The Department of Ophthalmology, Duke University Eye Center, Durham, NC

## Abstract

**Purpose:**

Myopia, or nearsightedness, is highly prevalent in Asian countries and is considered a serious public health issue globally. High-grade myopia can predispose individuals to myopic maculopathy, premature cataracts, retinal detachment, and glaucoma. A recent study implicated zinc finger protein 644 isoform 1 (*ZNF644*) variants with non-syndromic high-grade myopia in a Chinese-Asian population. Herein we focused on investigating the role for *ZNF644* variants in high-grade myopia in a United States (US) cohort.

**Methods:**

DNA from a case cohort of 131 subject participants diagnosed with high-grade myopia was screened for *ZNF644* variants. Spherical refractive error of -≤-6.00 diopters (D) in at least one eye was defined as affected. All coding, intron/exon boundaries were screened using Sanger sequencing. Single nucleotide allele frequencies were determined by screening 672 ethnically matched controls.

**Results:**

Sequencing analysis did not detect previously reported mutations. However, our analysis identified 2 novel single nucleotide variants (c.725C>T, c.821A>T) in 2 high-grade myopia individuals- one Caucasian and one African American, respectively. These variants were not found in normal controls. A rare variant - dbsSNP132 (rs12117237→c.2119A>G) - with a minor allele frequency of 0.2% was present in 6 additional cases, but was also present in 5 controls.

**Conclusions:**

Our study has identified two novel variants in *ZNF644* associated with high-grade myopia in a US cohort. Our results suggest that *ZNF644* may play a role in myopia development.

## Introduction

Myopia is a common ocular disorder resulting from excessive axial elongation of the globe [1,2]. High myopia in particular can predispose one to several ocular complications such as myopic maculopathy, premature cataracts, retinal detachment, and glaucoma, and is considered a public health concern in numerous countries around the world [3]. Myopia prevalence rates vary world-wide. A recent study by Vitale et al. [4] reported that 33.1% of the USA population greater than the age of 20 has some degree of myopia. Studies have shown that prevalence rates in Asia countries are higher. For example, 84% of school children in Taiwan develop myopia by the age of 18 [5,6]. Additionally, more than 85% of Hong Kong Chinese school children aged between 13 and 15 years are myopic [7]. The economic impact of refractive error management can be substantial- vision impairment correction costs account for 3.8 to 7.2 billion dollars annually in the USA alone [8].

Accepted as a common complex disorder, myopia is thought to be influenced by both genetic and environmental factors [9,10]. To date, over 20 genetic loci have been mapped using linkage analysis for both common myopia and high myopia [11-17]. A recent plethora of genome wide association studies have shown positive associations with refractive error [14,18-25]. Of late, large parallel sequencing techniques have been used to identify causal genes for ocular disorders including myopia. Rare ocular diseases such as retinitis pigmentosa and familial exudative vitreoretinopathy were among the first where causative genes were successfully identified using exome sequencing [26-28]. More recently, Mordechai et al. [29] identified a leprecan-like 1 (*LEPREL1*) mutation as causal for autosomal recessive high-grade axial myopia in a large, consanguinous Bedouin Israeli kindred. The mutation identified in their study demonstrated an autosomal recessive mode of inheritance with variable expressivity. As the lone finding for high myopia and autosomal dominant inheritance, Shi et al. [30] recently used exome sequencing to identify mutations in zinc finger protein 644 isoform 1 (*ZNF644*) – first in a large pedigree with autosomal dominant high myopia, and then replicated in a Chinese cohort. *ZNF644* is a transcription factor gene, and is expressed in the retina and retinal pigment epithelium (RPE) [30]. To our knowledge, the role of *ZNF644* has not been studied in a myopic USA cohort composed primarily of Caucasians. We therefore screened for *ZNF644* mutations in a high-grade myopia USA population data set. The identification of mutations in *ZNF644* in other ethnicities supplements existing knowledge of the etiology and heterogeneity of this debilitating eye disorder.

## Methods

### Patient information

Informed consent was obtained from all participants before entering the study, with approval by the Institutional Review Board according to the principles of the Declaration of Helsinki. High-grade myopia cases were defined as individuals with a spherical refractive error of greater than or equal to −6.00 diopters (D) in at least one eye. From venous blood samples, genomic DNA was extracted using AutoPure LS^®^ DNA Extractor and PUREGENE™ reagents (Gentra Systems Inc., Minneapolis, MN). DNA for 672 ethnically matched Caucasian healthy control participants was purchased commercially (The Centre for Applied Genomics, The Hospital for Sick Children, Toronto, Canada). Although the controls had no documented ocular malformations, refractive error information was not available for these individuals. Additionally, 50 ethnically matched African American participant controls with refractive error data were ascertained, and blood for DNA extraction was collected for this study.

### PCR and sequence analysis

*ZNF644* (NM_201269.1) encodes for a zinc finger transcription factor which maps to chromosome 1p22.2 (Chromosome1:91,380,860–91,487,671 – GRCh37.p5). The gene comprises 6 exons, five of which are coding. Polymerase chain reaction (PCR) and sequencing primers were designed to cover all coding and untranslated gene regions (UTR) including intron-exon boundaries using the ExonPrimer program (Helmholtz Center, Munich, Germany). Primers were selected to produce amplification products not to exceed 850 base pairs (bp) in size for optimal sequence output and analysis. A total of thirteen primer sets were designed to ensure full coverage of the exons and the flanking intronic regions (Table 1).

**Table 1:** Primers for PCR and sequencing of *ZNF644*.

***ZNF644* exon**	**Forward**	**Reverse**	**Product size (base pair)**
Exon 1	AAAATGCGTCCTTTTGGATG	GGAGGTGACCTTGTTTGGTT	492
Exon 2	AATGATGGTATTCTGGTTG	AAGTCAATTATTTGCATTTC	363
Exon 2*	ATCAGACCTGGAGAGGCAAA	TAGTCACATGAAGCCGAGCA	353
Exon 3.1	TCTGTGGTGTAGACAGCTGAA	TTGTATACATGACGTATTGGACTGTT	697
Exon 3.2	CTTTTTGGGGATCCCAGTTT	ACGTTGACTCTGCCTGAAGAA	580
Exon 3.3	TGAAAGTAGCAGGTGACTCAGAA	GTGGATCAGCCAACAACAGA	778
Exon 3.4	CAGGTTCTTCAAGGATGTCATTT	TGTGGAGAAGAGAGTTCACCTG	796
Exon 3.5	TTCTTTTCAGCAGAATTAAGTTTTTG	AGCACACGGAGTACTTGCATT	742
Exon 3.6	AAACTGACCACCCTAAAATGAGTT	TGGAGGGGAAGACTTGGATA	759
Exon 4	GCAGCTTAAACAGGAAGATTGTG	GAATTAACTCATTTTAGGGTGGTCA	790
Exon 5	TTTTAAGCCTATCTCCAAAAGTTCA	GAATGCATGCTTCAGGGAAT	395
Exon 6.1	TTAAAAACACATCTTCCACCCTA	TGAATTGGGAGTTTTGATGTTT	564
Exon 6.2	CGTCTATTCTAAACTGTGTAGTGAGCA	ACAGTGACATCAGAGCAAATTGA	829
Exon 6.3	CATTATATTGACCAATGAGGTGATTC	TGCTTACAGGACAGGTTTGC	782

*Denotes isoform 2 of exon 2, which is an untranslated exon.

Samples were amplified using standard PCR protocol and amplicons were visualized after agarose gel (2%) electrophoresis. Sequencing of the amplicons was then completed on an Applied Biosystems ABI3730 xlrobotics using BigDye™ Terminator 3.1 technology (Applied Biosystems, Inc. [ABI], Foster City, CA). Sequences were analyzed using the Sequencher 5.0™ program (Gene Codes, Ann Arbor, MI), and were compared against the known reference sequence (GRCh37.p5) and analyzed for sequence variation. Single nucleotide variants (SNVs) that were novel and/or coding non-synonymous with a minor allele frequency (MAF) of less than 1% were checked for co-segregation in remaining family member samples.

### Genotyping

Allelic discrimination assays were employed to measure the allelic frequencies in 672 Caucasian matched control DNA samples using the TaqMan® SNP Genotyping system (Applied Biosystems). Assays were designed according to Applied Biosystems specifications using a combination of unlabeled primers and minor groove binding (MGB) probes with fluorescently labeled dyes (FAM and VIC) to interrogate the base pair of interest. Reactions were completed and ABI 7900 robotics was used to read the allelic calls for each control sample (Applied Biosystems). SDS v2.4 Software provided by ABI was used to analyze each sample and accurately analyze each genotype call.

## Results

Full ophthalmologic exams were performed on all subject participants with one or more individual(s) with high grade myopia. The average cycloplegic spherical refractive error for 131 high myopia cases was −11.22 D for the right eye (OD) and −11.48 for the left eye (OS), and the range was from −6.00 D to −50.00 D (OD) and −5.25 D to −50.00 D (OS) across all cases. Of the 131 case participants screened for *ZNF644*, 74% (97/131) were Caucasians, 12.2% (16/131) were African Americans, 10% (13/131) were Asians, while 5 individuals (3.8%) were of Hispanic descent or declined to self-identify.

The entire coding and untranslated DNA sequence of *ZNF644* was sequenced in 131 high myopia patients. In all, we identified a total of 31 heterozygous SNVs in *ZNF644*, of which 10 were missense, 7 silent, 9 untranslated, and 5 intronic (Table 2 and Table 3).

**Table 2:** Summary of affected patients with *ZNF644* variants in 131 high-grade myopia cases.

**Family**	**Individual**	**Gender**	**Ethnicity**	**Spherical Refractive Error (D)**		**Exon**	**Amino Acid Change**	**cDNA Position**	Chromosome 1 base pair location*	**Variant Identification†**	**Co-segregation**
** **	** **	** **	** **	**OD**	**OS**	** **	** **	** **	** **	** **	** **
MYP104	IND0603564	M	African American	−18.00	−18.00	3	T242M	c.725C>T	91406186	Novel	N/A
MYP163	IND0603809	F	Caucasian	−7.50	−6.75	3	E274V	c.821A>T	91406090	Novel	N/A
MYP8	IND0519772	F	Asian	−11.00	−12.25	3	H706Y	c.2116C>T	91404795	Novel	NO
MYP19	IND0519791	M	Caucasian	−19.25	−21.00	3	K707E	c.2119A>G	91404792	rs12117237	YES
MYP83	IND0603414	M	Caucasian	−9.75	−9.00	3	K707E	c.2119A>G	91404792	rs12117237	NO
MYP89	IND0603458	M	Caucasian	−6.00	−6.00	3	K707E	c.2119A>G	91404792	rs12117237	YES
MYP102	IND0603552	F	Caucasian	−16.00	−18.00	3	K707E	c.2119A>G	91404792	rs12117237	N/A
MYP113	IND0603616	F	Caucasian	−12.75	−13.50	3	K707E	c.2119A>G	91404792	rs12117237	NO
MYP129	IND0603676	F	Caucasian	−5.50	−6.25	3	K707E	c.2119A>G	91404792	rs12117237	N/A
MYP2	IND0519764	M	Caucasian	−10.75	−10.50	4	R1100H	c.3299G>A	91403431	Novel	NO

Rare is defined by variants not found in controls or have minor allele frequency of less than 1% . Abbreviations: N/A- not available, OD- right eye, OS- left eye, DS- Diopter Sphere, M- Male, F- Female, *UCSC hg19 browser – GRCh37.p5, †-dbSNP132.

**Table 3:** Summary of variants identified in *ZNF644* in 131 high-grade myopia cases.

Chromosome 1 base pair location*	**Variant type**	dbSNP132	**Allele change**	**Amino acid change**	**Population**
91487710	UTR	Novel	A>G	N/A	Caucasian
91487657	UTR	Novel	C>T	N/A	Caucasian
91487013	UTR	Novel	G>T	N/A	Caucasian
91447985	Intronic	rs358691	A>G	N/A	Caucasian
91406677	Synonymous	rs17131243	G>A	L78L	African American
91406033	Synonymous	Novel	G>A	R293Q	Caucasian
91405699	Synonymous	rs41286763	C>T	T404T	Caucasian
91405245	Nonsynonymous	rs17131242	A>G	M556V	African American^¥^
91405215	Nonsynonymous	rs60262072	A>T	T566S	African American^¥^
91404592	Synonymous	Novel	C>T	H773H	Hispanic
91404532	Synonymous	Novel	C>T	D793D	African American
91404530	Nonsynonymous	rs10922938	C>T	A794V	African American^¥^
91404303	Nonsynonymous	rs59922637	A>G	T870A	African American^†^
91404256	Nonsynonymous	rs41286761	G>T	E885D	Caucasian
91383756	Intronic	Novel	A>G	N/A	Caucasian
91383589	Intronic	rs2448020	G>T	N/A	Multiple
91382635	Intronic	rs17131234	G>C	N/A	African American
91382406	Synonymous	rs114618312	C>T	A1311A	African American
91382370	Synonymous	Novel	C>T	A1323A	Asian
91382086	UTR	Novel	A>T	N/A	African American
91381679	UTR	rs1188952	C>T	N/A	Multiple
91381534	UTR	Novel	A>C	N/A	Caucasian
91381240	UTR	Novel	C>T	N/A	Caucasian
91381181	UTR	Novel	G>C	N/A	African American
91381105	UTR	rs17131232	A>T	N/A	Multiple
91380797	Intronic	rs115299241	C>T	N/A	African American

Abbreviations: N/A- not applicable, UTR- untranslated region, Multiple - variant found in more than one population, dbSNP132. *GRCh37.p5. ¥-Identified in IND0603564. †-Identified in IND0603416.

From seven synonymous sequence variants detected in our cohort, we identified 4 novel SNVs in four separate individuals, which were either within exon 3 or 6. We also found 9 SNVs in the UTR, where 6 of 7novel variants were unique to an individual. In addition to the exonic variants, 4 intronic variants were previously reported in the dbSNP132 database, while 1 was novel and unique to an individual. All intronic variants observed had high MAFs, and/or were not located near splice-site junctions (Table 2).

**Figure 1:**
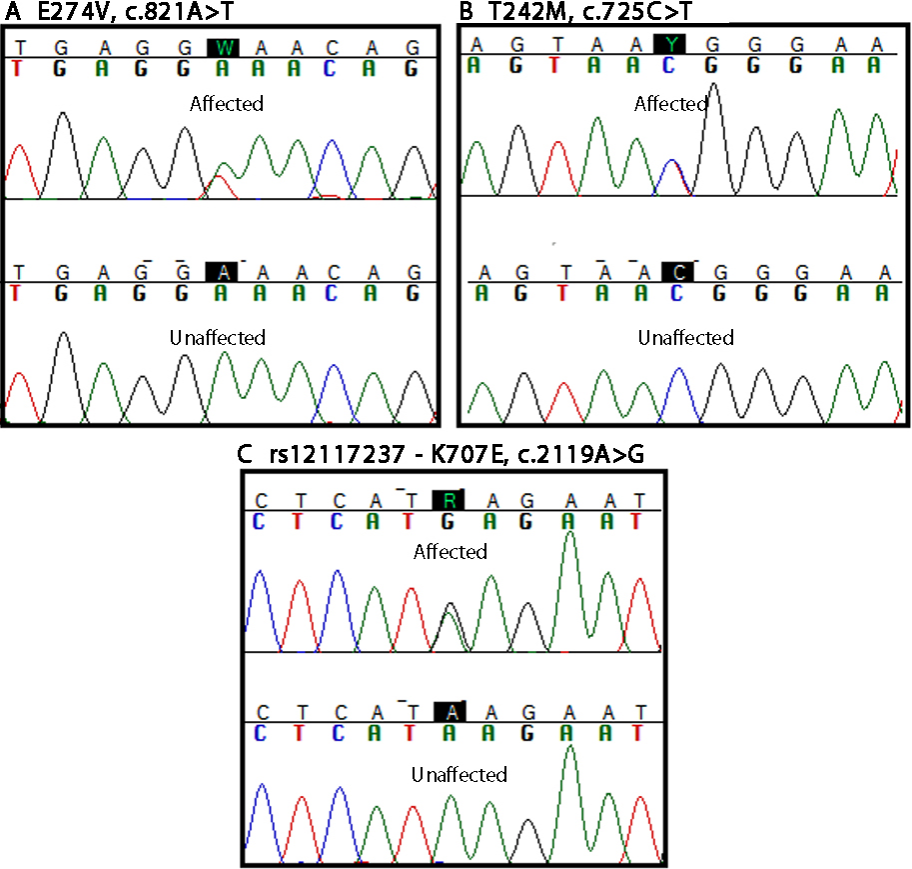
Sequence chromatogram of identified *ZNF644* variants. Base pair location in bold depicts the variant change in affected individual compared to an unaffected individual. **A**: Novel variant identified in individual MYP0603809 with E274V (c.821A>T) change. **B**: Novel variant identified in individual MYP0603564 with T242M (c.725C>T) change. **C**: rs12117237 variant (K707E, c.2119A>G) that was present in 5 high myopic cases.

Of the 10 missense variants identified, 4 were novel and 6 were reported in the dbSNP132 database (Table 3). One novel missense variant was identified in Caucasian individual IND0603809. This adenine to thymine substitution at position c.821A>T alters glutamic acid to valine (Glu274Val), and was not found in other high myopic case samples. A second novel missense variant was identified on exon 3 in African American individual IND0603564. This cytosine to thymine substitution (c.725C>T) results in a threonine to methionine (Thr242Met) amino acid change was only present in this individual. Due to the lack of additional family members for these individuals, segregation analysis was not possible (Figure 1). Novel variants c.2116C>T (His706Tyr) and c.3299G>A (Arg1100His) were identified in Caucasian individuals IND0519772 and IND0519764, respectively. Neither variant segregated with disease after screening available family members.

To confirm the rarity of c.821A>T, a TaqMan genotyping assay was employed to screen a DNA sample data set of Caucasian controls. The variant (c.821A>T) was not replicated in 672 Caucasian controls by allelic discrimination genotyping method. The novel variant found in African American individual IND0603564 (c.725C>T) was not replicated in 50 African American control samples via Sanger sequencing (Figure 1).

**Figure 2:**
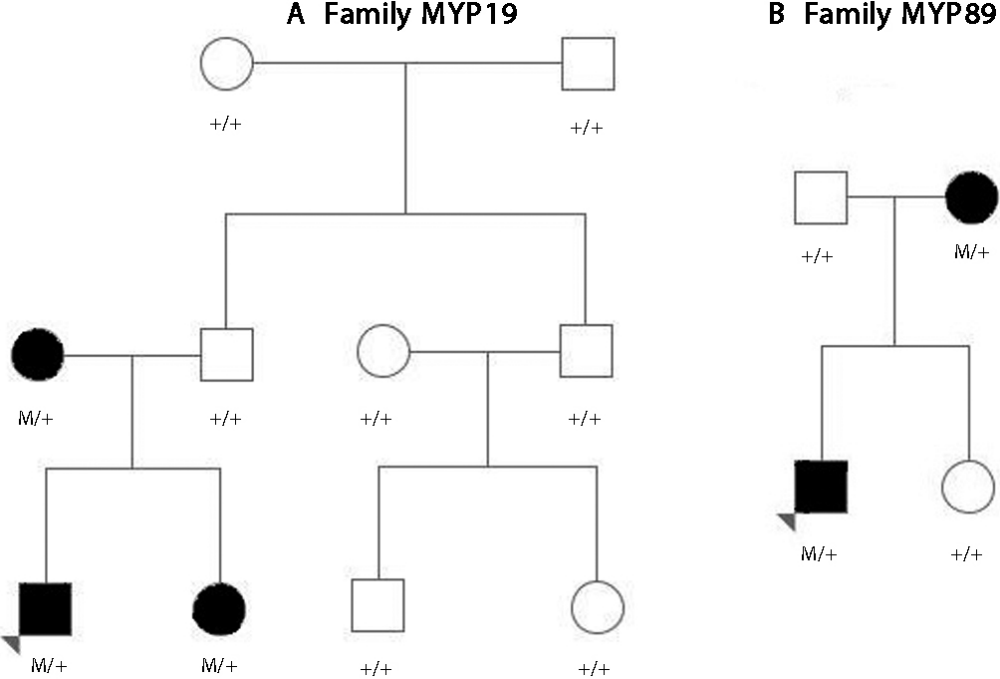
Pedigree and segregation of rs12117237 on MYP19 and MYP89 family. **A**: Kindred structure and segregation of *ZNF644* rs12117237 in MYP19. **B**: Kindred structure and segregation of *ZNF644* rs12117237 in MYP89. Affected individuals are identified by solid squares (male) or circles (females). Normal individuals are identified by open symbols. Colored triangle depicts index case patient. M: 707E recessive allele of *ZNF644*; +: K707 normal allele of *ZNF644*.

Four missense variants in dbSNP132 were identified in African American individuals IND0603564 and IND0603416 but were ruled out due to minor allele frequency not meeting criteria below 1% (Table 3). Missense variant rs12117237, the only variant in dbSNP132 with a minor allele frequency of less than 1% in public databases was identified in 6 Caucasian high myopia cases (0.2%). The minor allele was also seen in 0.75% (5/672) of controls. Fisher’s exact two-sided test for rs12117237 demonstrated a p-value of 0.0015 when comparing the prevalence rates of the allele between cases and controls (SAS Institute Inc., Cary, NC). Other family members of the cases were sequenced to determine co-segregation. Two cases had uninformative family members or were simplex cases, and thus were not useful for determining segregation of the variant. Of the 6 myopia cases identified with rs12117237, 4 families (MYP19, MYP83, MYP89, and MYP113) had at least 3 additional family members. These members were sequenced, and MYP19 and MYP89 demonstrated phenotype co-segregation with the SNV (Figure 2).

## Discussion

Mutation screening of *ZNF644* has successfully identified 2 novel missense variants (c.725C>T, c.821A>T) in two ethnicities within our US population, thus supporting the original report of a causal candidate gene for high-grade myopia (Table 2). The two novel variants from our study (c.725C>T, c.821A>T) localize to a conserved region on the third exon, where Shi et al. [30] previously identified several mutations. The variants were not seen in 1344 and 100 ethnically matched chromosomes, respectively, confirming their rarity. PolyPhen-2 software predicted c.725C>T to be possibly damaging, while c.821A>T was tolerated. With the additional variant information from our study, exon 3 may be a hotspot for susceptibility for high-grade myopia in multiple ethnicities. The clustering of variants in exon 3 may depict the importance in protein domain structures and gene regulatory functions of ZNF644.

The single nucleotide variant rs12117237 was present in 6 Caucasian case samples, but also present in 5 ethnically matched control DNA samples. Public database dbSNP132 reports this variant’s MAF to be 0.2% in a European population, and our p-value of 0.0015 by Fisher’s exact test suggests presence of the alleles likely did not occur by chance. As a result of sequencing additional family members in three high myopia cases for the variant, we discovered 100% co-segregation of the mutation with the disease phenotype in two of four families. PolyPhen-2 software predicts the amino acid change to be tolerated. The variant could be a common rare variant, and proper functional validation would be important to determine whether it is a risk or a causal allele [31]. However, the MAF is in line with prevalence rates for high-grade myopia in a general population and a founder mutation effect may be plausible. Clinical information was missing refractive error from the 5 ethnically matched controls who also presented with the variant in the heterozygous state. The prevalence rate of high myopia is estimated to be 4.5% in the US population [32,33]. Moreover, a recent study suggests that the rarer a variant, the higher the likelihood that the variant functionally alters the protein – thus rs12117237 may be associated with high-grade myopia in a Caucasian population [34]. A larger case-control population data set should be tested to understand the true significance of the association of this variant to high-grade myopia in Caucasians.

ZNF644 is a transcription factor that may play a role in protein domain structures or regulatory functions [30]. Expressed in all tissue types, it follows the trend of ubiquitously expressed genes pathogenic to ocular diseases [35]. To date, it is widely accepted that transcription factor genes in both humans and mouse can play an important role in mammalian eye growth and development. For example, paired-like homeodomain transcription factors 2 and 3 (*PITX2*, *PITX3*) have been implicated in Axenfeld-Rieger syndrome and cataracts, respectively [36-38]. Microphthalmia transcription factor (*Mitf*) is associated with ocular albinism, and paired box 6 (*PAX6*) has been associated with retinal degeneration, extreme myopia, and corneal innervation [20,39-46]. However, the true function of *ZNF644* remains unclear, and molecular characterization of *ZNF644* is necessary.

To the best of our knowledge, this is the first successful study confirming a gene implicated with non-syndromic high-grade myopia determined by exome sequencing. We identified two novel variants in *ZNF644* in our cohort, in addition to a known variant that demonstrated association. The discovery of previously unidentified variants is not expected due to genetic heterogeneity present in rare complex disease across multiple populations [47]. Ethnic group specific alleles due to founder effect may explain why the previously reported variants were only present in the Asian population whereas the variants discovered in this report appear to be specific to the Caucasian and African American ethnicities. Determining pathogenic rare missense variants remains a challenge for complex diseases. Identification of novel variants in separate ethnicities emphasizes the importance and demonstrates the power of current research approaches.
